# Low-Temperature Reduction of Graphene Oxide: Electrical Conductance and Scanning Kelvin Probe Force Microscopy

**DOI:** 10.1186/s11671-018-2536-z

**Published:** 2018-05-08

**Authors:** Oleksandr M. Slobodian, Peter M. Lytvyn, Andrii S. Nikolenko, Victor M. Naseka, Oleg Yu. Khyzhun, Andrey V. Vasin, Stanislav V. Sevostianov, Alexei N. Nazarov

**Affiliations:** 1 National Technical University of Ukraine “Igor Sikorsky KPI”, 37, Prosp.Peremohy, Kyiv, 03056 Ukraine; 2 V. Lashkaryov Institute of Semiconductor Physics NAS of Ukraine, 41 Prosp. Nauki, Kyiv, 03028 Ukraine; 3 Frantsevych Institute for Problems of Materials Science NAS of Ukraine, 3 Krzhizhanovsky St., Kyiv, 03680 Ukraine; 4 Chuiko Institute of Surface Chemistry NAS of Ukraine, 17 Generala Naumova St, Kyiv, 03164 Ukraine

**Keywords:** Graphene oxide, Reduced graphene oxide, Electrical resistivity, Atomic force microscopy, Scanning Kelvin probe force microscopy, Raman spectroscopy, FTIR spectroscopy

## Abstract

Graphene oxide (GO) films were formed by drop-casting method and were studied by FTIR spectroscopy, micro-Raman spectroscopy (mRS), X-ray photoelectron spectroscopy (XPS), four-points probe method, atomic force microscopy (AFM), and scanning Kelvin probe force (SKPFM) microscopy after low-temperature annealing at ambient conditions. It was shown that in temperature range from 50 to 250 °C the electrical resistivity of the GO films decreases by seven orders of magnitude and is governed by two processes with activation energies of 6.22 and 1.65 eV, respectively. It was shown that the first process is mainly associated with water and OH groups desorption reducing the thickness of the film by 35% and causing the resistivity decrease by five orders of magnitude. The corresponding activation energy is the effective value determined by desorption and electrical connection of GO flakes from different layers. The second process is mainly associated with desorption of oxygen epoxy and alkoxy groups connected with carbon located in the basal plane of GO. AFM and SKPFM methods showed that during the second process, first, the surface of GO plane is destroyed forming nanostructured surface with low work function and then at higher temperature a flat carbon plane is formed that results in an increase of the work function of reduced GO.

**Electronic supplementary material:**

The online version of this article (10.1186/s11671-018-2536-z) contains supplementary material, which is available to authorized users.

## Background

Graphene and graphene-based materials have very attractive physical and optical properties [1–3] which can be employed into a lot of applications such as nanoelectronics [4], chemical and biosensors [5, 6], solar-cells [7], effective catalysts [8], and supercapacitors [9, 10]. A necessity of inexpensive mass production of these materials directed the interest of a great army of researchers into the study of graphene oxide (GO) reduction [11] which allows obtaining a graphene material with needed properties using chemical [12] or radiation [13] methods. One of the simplest reduction techniques is the thermal one which is usually performed in vacuum to desorb oxygen molecules from carbon π bonds [11]. However, there are some papers which demonstrate the GO reduction at ambient conditions at relatively low temperatures that results in considerable decreasing of the material resistance [14, 15], and that, of course, is very attractive for different applications. Up to now, the relationship between significant changes in conductivity at low temperature with other parameters of the film and their stability during a long time is controversial. This paper analyzes the changes of electrical resistance of GO during the thermal reduction in air and associate it with the results obtained from atomic force microscopy (AFM), scanning Kelvin probe force microscopy (SKPFM), micro-Raman spectroscopy (mRS) and with changing of GO’s chemical bonds measured by FTIR spectroscopy and X-ray photoelectron spectroscopy (XPS).

## Methods/Experimental

### Sample Preparation

The GO was synthesized by Hummers’ method [16] and transformed into water solution. A main chemical composition of the synthesized GO material and annealed at 50 °C was determined using XPS. The carbon/oxygen ratio was found to be 2.31 in pristine GO which is in agreement with values reported for similar oxidation processes [17, 18]. The C1s XPS spectrum of GO clearly indicates a considerable degree of oxidation with four components that correspond to carbon atoms in different functional groups: 52.6% of the non-oxygenated C in sp^3^/sp^2^ state (284.7 eV), 26.6% of the C in C–O bonds (286.7 eV), 11.5% of the carbonyl carbon (C=O, 287.6 eV), and 8.3% of the carboxylate carbon (O–C=O, 289.0 eV) [19].

The water dispersion was drop-casted using a micropipette onto both glass and silicon substrates at substrate temperature of about 50 °C. The samples at silicon substrates were used for IR spectra measurements. To perform scanning Kelvin probe force microscopic measurements and XPS ones, the Ni/Si structures were fabricated where Ni film was deposited by DC magnetron sputtering method. Thermal reduction of the samples was performed in the temperature range from 100 to 250 °C (15 min) in ambient atmosphere.

### Methods of Measurement

Thermally activated desorption in the GO was characterized by thermogravimetry (TG) at atmospheric conditions with the use of a derivatograph Q-1500D apparatus (Paulik and Erdey). Chemical bonds in the GO film deposited on silicon wafer were detected by FTIR spectroscopy using Bruker Vertex 70 V spectrometer and XPS using the UHV-Analysis-System (SPECS Surface Nano Analysis Company) possessing the residual pressure less than 5 × 10^− 10^ mbar and equipped with a PHOIBOS 150 energy analyzer. The XPS spectra of the rGO films were excited by an X-ray Mg Kα source (*E* = 1253.6 eV) and were recorded at constant pass energy of 35 eV. Low-energy electrons emitted by a flood gun were employed to overcome charging effects.

Micro-Raman measurements were carried out at room temperature in backscattering configuration using a triple Raman spectrometer, T-64000 (Horiba Jobin Yvon), equipped with an electrically cooled CCD detector. The 488 nm line of an Ar–Kr ion laser was used for excitation. Exciting radiation was focused on the sample surface with a × 50 optical lens giving a laser spot size of about 1 μm (diameter). The laser power on the sample surface was always kept below 1 mW to avoid laser heating effects or damage.

Resistivity was measured with the four-point probes (4PP) method [20]. Surface morphology and surface potential of the GO flakes were measured correspondingly by AFM and SKPFM using a NanoScope IIIa Dimension 3000 scanning probe microscope. The two-path frequency modulated SKPFM technique was used. First, a surface profile was obtained. Then, the tip was lifted up to 20 nm, and the electrostatic tip–surface contact potential difference was measured along the previously captured surface profile. Lift height was selected large enough to avoid the van der Waals tip–surface interaction and small enough to keep highest resolution and sensitivity of frequency modulated KPFM. KPFM maps transformations were estimated also for height of 40 nm (Additional file 1: Figure S1) as well as for the cases of sample and tip grounded (see Additional file 1: Figure S2). In last case, the predictable losses of resolution and sensitivity were observed on different graphene flakes, and no principal changes were detected. Measurements were carried out using an EFM 20 (NanoWorld) Si probe covered with Pt/Ir film. The SKPFM method permits the mapping of phase-inhomogeneous surfaces by measuring and nullifying electrostatic tip–surface interaction controlling dc potential on the tip [21].

In order to study a thermal reduction of a definite GO flake a special sample heating holder was fabricated on which the samples could be subjected to thermal heating from 80 to 230 °C out of the measurement system, and returned back after cooling to room temperature. Additionally, the GO flakes were deposited on Ni surface which was grounded to avoid the electrostatic charge, and in order to provide more accurate measurements of contact potential difference (CPD) regarding to Ni.

## Results and Discussion

### Thermogravimetry

**Fig. 1:**
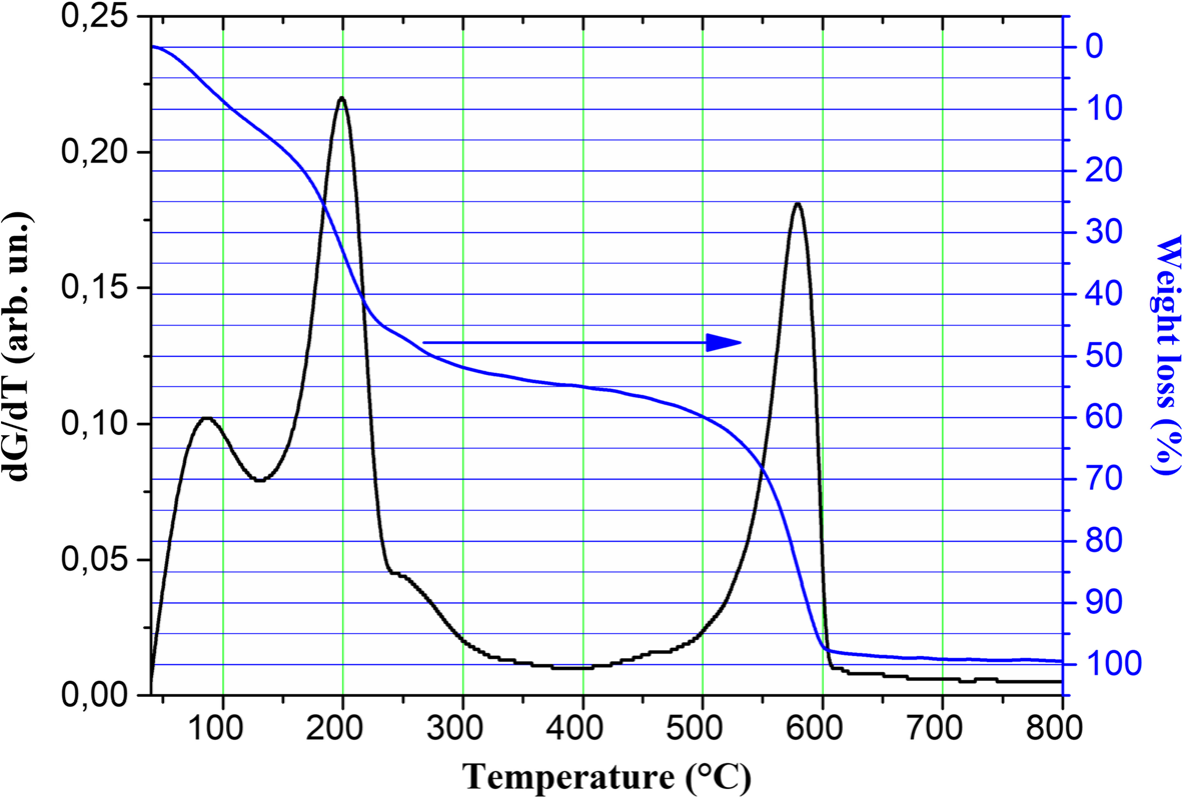
Weight loss during the GO reduction process in the temperature range 40–800 °C. Heating rate is 10 °C/min. dG/dT ratio is also shown

Measurements of weight loss during heating of the GO with rate of 10 °C/min attested that 50% of all weight was lost in the temperature range below 300 °C (Fig. 1). Up to 500 °C, the sample loses additional 10% of weight and 37% of weight is lost in the range from 500 to 600 °C (Fig. 1). It was shown [22] that weight loss in the range from 500 to 700 °C in air atmosphere is mainly associated with combustion of the carbon skeleton. The weight loss under 200–250 °C is determined by molecular water desorption up to 150 °C and then by oxygen desorption from epoxy or alkoxy (C–O–C) groups located in the graphite sheet [23, 24].

### FTIR Spectroscopy and XPS

**Fig. 2:**
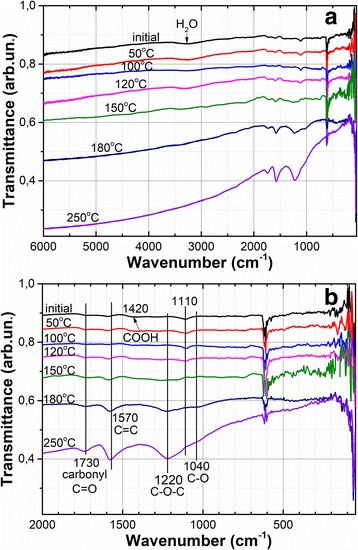
FTIR spectra as a function of annealing temperature in the range of wave numbers from 100 to 6000 cm^−1^ (**a**) and from 100 to 2000 cm^−1^ (**b**)

The FTIR spectra of the initial GO films demonstrate the appearance of OH bonds (Fig. 2). The absorption band centered at 3300 cm^− 1^ corresponds to the stretching mode of OH bonds from C–OH group or water molecules [25]; a band at 1420 cm^− 1^ is probably associated with the stretching mode of COOH group [26]; a band at 1110 cm^− 1^—OH groups from alcohol [27]. After annealing at 180 °C for 15 min, all observed OH bonds were not registered (see Fig. 2b).

After annealing at 180 °C stretching mode at 1220 cm^− 1^, corresponding to apoxy (C–O–C) groups, and at 1050 cm^− 1^, corresponding to alkoxy (C–O–C) groups [25] are formed. Additionally, a band at 1730 cm^− 1^, which is associated with stretching mode of carbonyl groups (C=O) at the edges of the GO flakes [25], increases in amplitude. It should be noted that increase of a peak with the maximum at 1570 cm^− 1^ corresponding to vibration of C=C groups (skeleton vibration of the graphene plane [26]) attests the formation of non-oxidized graphite regions. Formation of inefficient band with the maximum at 450 cm^− 1^ can be linked with generation of amorphous carbon nanoclusters [28] in the GO film during the annealing.

Three well expressed absorption bands are observed in the FTIR spectrum after annealing at 250 °C. These are the stretching mode of carbonyl groups (1730 cm^− 1^), the stretching mode of epoxy groups (1220 cm^− 1^), and the vibration of C=C groups (1570 cm^− 1^). The first mentioned mode attests the high temperature desorption of carboxyl groups located at the edges of the GO flakes, and the increased amplitude of the last mentioned mode indicates the increase in dimensions of unoxidized graphene areas. Moreover, the IR spectrum after annealing at 250 °C demonstrates strong adsorption in the range from 2000 to 6000 cm^− 1^ (Fig. 2a) that is associated with absorption of free electrons [29] and is in agreement with the considerable increase in electrical conductivity of the GO films after the annealing.

**Fig. 3:**
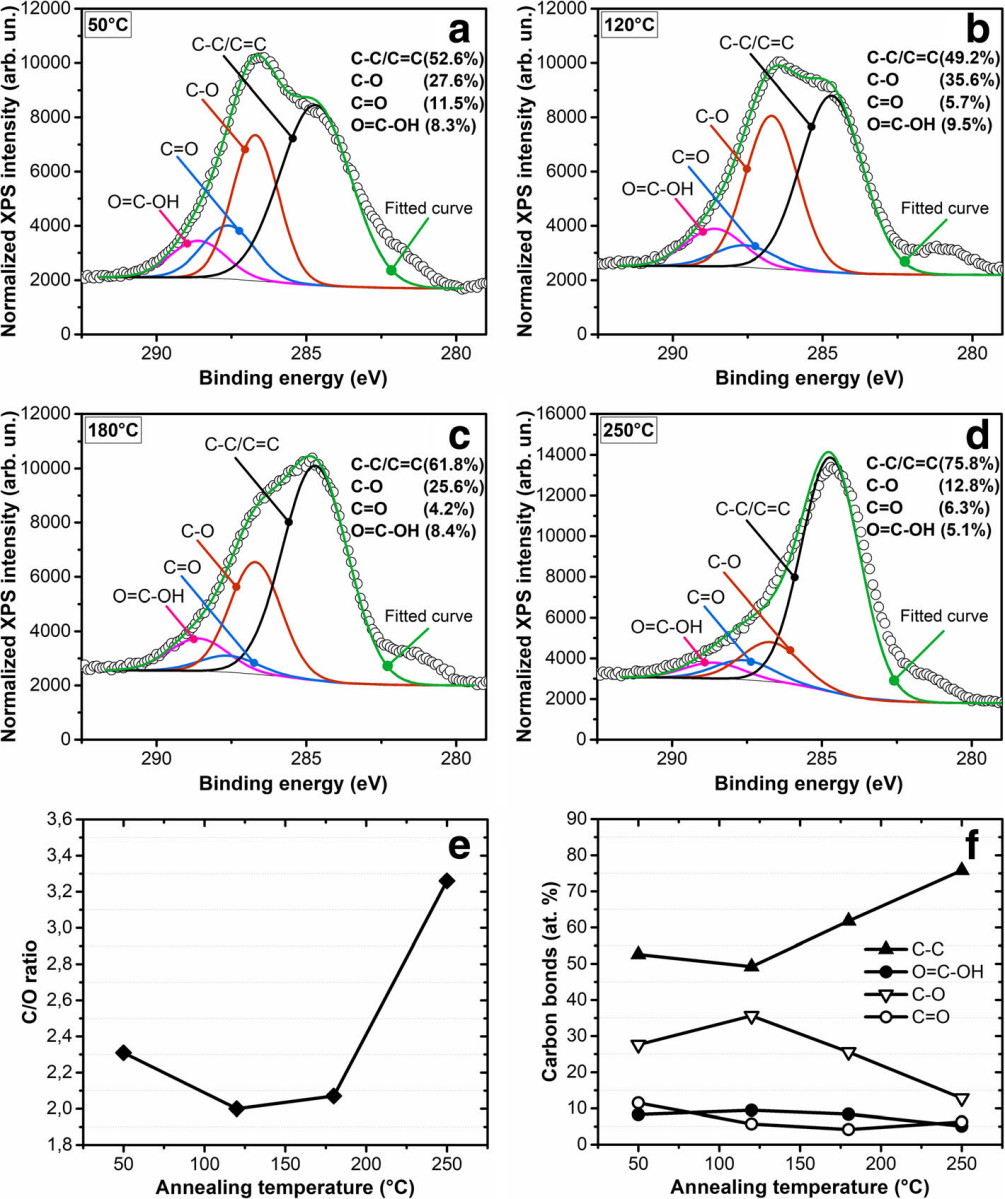
C 1 s XPS spectra (*hν* = 1253.6 eV) collected on GO thin film deposited on Ni(100 nm)/Si and annealed in air for 15 min at the temperatures 50, 120, 180, and 250 °C (**a**–**d**). The different components related to various chemical shifts of carbon bonds are indicated. The relation of areas of the C1s to O1s XPS peaks (**e**) and the atomic percentages of different carbon bonds identified by XPS as a function of annealing temperature (**f**)

A chemical composition of the GO during the restoration can be estimated quantitatively employing the XPS method. Curve fitting of the XPS spectra was performed using a Gaussian-Lorentzian peak shape after a Shirley background correction (Fig. 3a–d). Only one peak was used to fit the graphitic (C=C) and aliphatic (C–C) carbon atoms due to the close proximity of their binding energies [30].

Oxidation level (ratio of carbon concentration to oxygen one) was estimated from a ratio of areas for C1s and O1s peaks (see Additional file 1: Figure S3). The C/O ratios were calculated in dependence on annealing temperature, and it was shown that at 50, 120, 180, and 250 °C, the relations were correspondingly 2.31, 2.00, 2.07, and 3.26 (see Fig. 3e). Thus, no oxygen desorption from GO film is observed in the range of the thermal annealing from 50 to 180 °C. Probably, desorption of molecular water in air atmosphere occurs together with oxygen trapping on carbon dangling bonds from air.

It should be noted that during the thermal annealing up to 180 °C transformation of carbonyl carbon bonds (C=O, 287.6 eV) into C–O bonds (286.7 eV) takes place while a concentration of the carboxylate carbon (O–C=O, 289.0 eV) remains almost constant (see Fig. 3f). The last bonds are usually formed in edges of GO flakes [12]. Further increase of annealing temperature results in increase of the non-oxygenated carbon concentration which at annealing temperature of 250 °C reaches 76% from total carbon concentration (C/O = 3.26) in the reduced GO. After thermal annealing at 250 °C small concentrations of carboxylate carbon, carbonyl carbon and C–O bonds are observed that totally corresponds to the results obtained by FTIR spectroscopy (see Fig. 2).

### Micro-Raman Scattering Spectroscopy

**Fig. 4:**
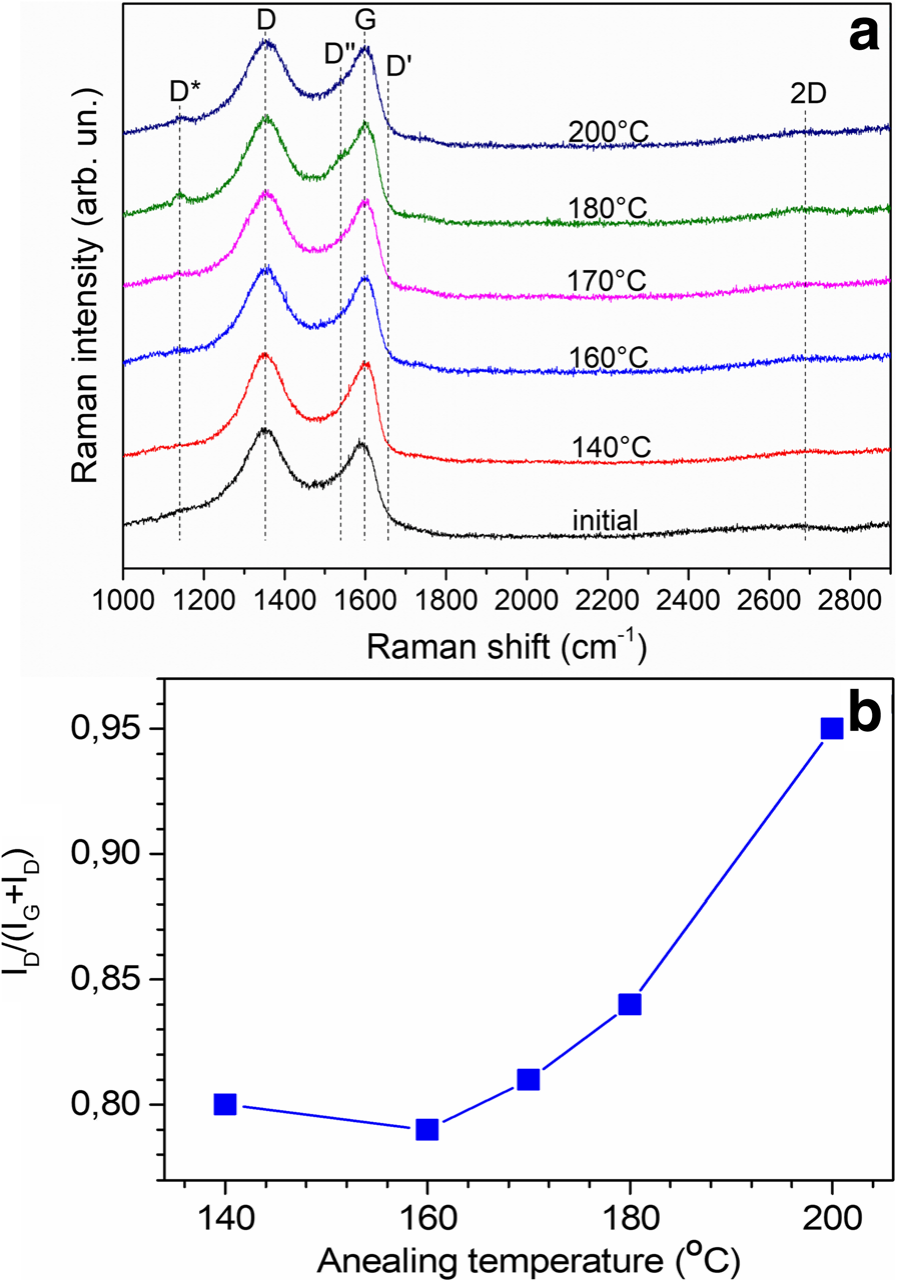
Normalized and y-shifted Raman spectra for rGO samples of different annealing temperatures (**a**). Dependence of *I*_*D*_/(*I*_*G*_ + *I*_*D*_) ratio on annealing temperature (**b**)

The micro-Raman spectra were recorded to characterize the GO microstructure. All the spectra are dominated by the D and G peaks centered at ~ 1350 and ~ 1590 cm^− 1^ and very weak 2D band centered at ~ 2700 cm^− 1^ (Fig. 4a). An important feature of the Raman spectra is the presence of the broad shoulder between G and D peaks. It was shown [31, 32] that Raman spectrum of GO can be described by five bands: G, D, and D’ (high-frequency shoulder of the G band) and two bands referred to as D* (~ 1150–1200 cm^− 1^) and D” (~ 1500–1550 cm^− 1^). Using approach proposed in [32], the spectra, presented in Fig. 4a, were fitted by five lines. Sample of the fitting is presented in Additional file 1: Figure S4. The normalized Raman spectra show that D* and D” lines increase in intensity with the annealing temperature and after annealing at temperature above 180 °C some sharp feature with maximum at ~ 1140 cm^− 1^ appears (Fig. 4a).

The nature of the D* and D” bands is controversial. It was showed by Ferrari and Robertson [33] that these two bands are the sum and difference of C=C stretching and CH wagging modes of trans-polyacetylene (alternating chain of sp^2^ carbons with a single hydrogen bonded to carbon) in nanocrystalline diamond and not due to sp^3^ carbons, that is an appearance of these bands is directly connected with hydrogen. However, in our case, as it was shown by our FTIR analysis (see previous section), a hydrogen in different bonding is desorbed from GO at temperatures below 180 °C. Also, it was early reported in ref. [34] that the D* line is actually associated with sp^3^ rich phase of disordered amorphous carbons, and in paper [31] it was suggested that these bands are due to finite size of crystallites and consequent increase in defects. Microcrystalline formation with defect generation is more suitable mechanism for our case. Interestingly, that similar feature at ~ 1140 cm^− 1^ was observed in cluster-assembled carbon thin films at 1180 cm^− 1^ [35] and was associated with microcrystalline or “amorphous” diamond phase. Moreover, the sharp peak at ~ 1140 cm^− 1^, which appears in addition to more broaden D* band and is clearly observed for the samples annealed at 180 and 200 °C, can be supposedly attributed to specific sp^3^-type defects, which are introduced in the desorption process at elevated temperatures. Similar sharp feature was observed for the covalently functionalized graphene and was attributed to trans-polyacetylene chains caused by the introduction of sp^3^ defect sites [36]. However, all these suggestions need additional experimental confirmation.

As it was shown in [32], the Cuesta model [37] correlating the nanocrystallites size (*L*_*a*_) with *I*_*D*_/(*I*_*D*_ + *I*_*G*_) ratio is more appropriate to characterize disorder in GO. Analysis of integrated peak intensities (Fig. 4b) showed, that the *I*_*D*_/(*I*_*D*_ + *I*_*G*_) ratio remains almost invariable at annealing temperature up to 160 °C and increases significantly at higher temperatures, thus reflecting increase in disorder of GO.

### Electrical Resistance of the GO Films

**Fig. 5:**
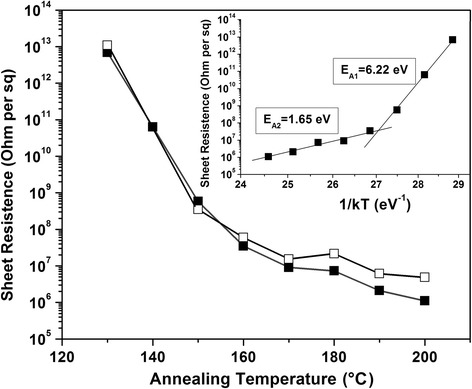
Sheet resistivity measured by 4PP method vs. annealing temperature in air ambient. Inset: Arrhenius plot. Black squares—initial measurements, empty squares—measurements after 6 months

The study of resistivity of the GO films by 4PP method shows that thermal heating of the samples in the temperature range from 100 to 200 °C (for 15 min) results in a decrease of the sheet resistance from 10^13^ to 10^6^ Ω/sq (Fig. 5). Taking into account the GO film thickness of about 40 nm (see AFM results in Additional file 1: Figure S3 s (a)) the resistivity equals to about 4 × 10^− 2^ Ω m which is low enough but much higher than the value for graphite (1 × 10^− 5^Ω m) [38].

The influence of annealing on resistivity of GO in this narrow temperature range can be described by two processes with activation energies of *E*_A1_ = 6.22 eV and *E*_A2_ = 1.65 eV (see inset Fig. 5). As it was shown by XPS measurements, no considerable reduction of the GO is observed during thermal annealing in air in range from 50 to 150 °C. Therefore, it can be suggested that the first activation energy is probably related to a complex process of interlayer water and OH groups desorption from the GO film (see Fig. 2b) which results in a strong decrease of distance between GO layers [39] improving electrical connections between flakes of different layers and considerably decreasing the resistivity of the GO film.

The second process related to decrease of the GO film’s resistivity is probably mainly determined by the desorption process of epoxy and alkoxy oxygen atoms together with carbon [40] and by formation of unoxidized graphene clusters [41]. The obtained activation energy is exactly the same as that obtained from resistivity measurements during thermal reduction in paper [14] and is very similar to values extracted by differential scanning calorimetric (DSC) method—1.47 eV in [21] and 1.73 eV in [40]. The difference can be associated with the experimental conditions.

To estimate the stability of obtained resistivity of the reduced GO (rGO) in air atmosphere, the measurements were repeated for the same samples after 6 months. The resistivity increases no more than twice for the annealing temperature range from 180 to 200 °C (empty squares in Fig. 5) that attests a good stability of the rGO structure obtained by low-temperature annealing in air ambient.

### AFM and SKPFM

**Fig. 6:**
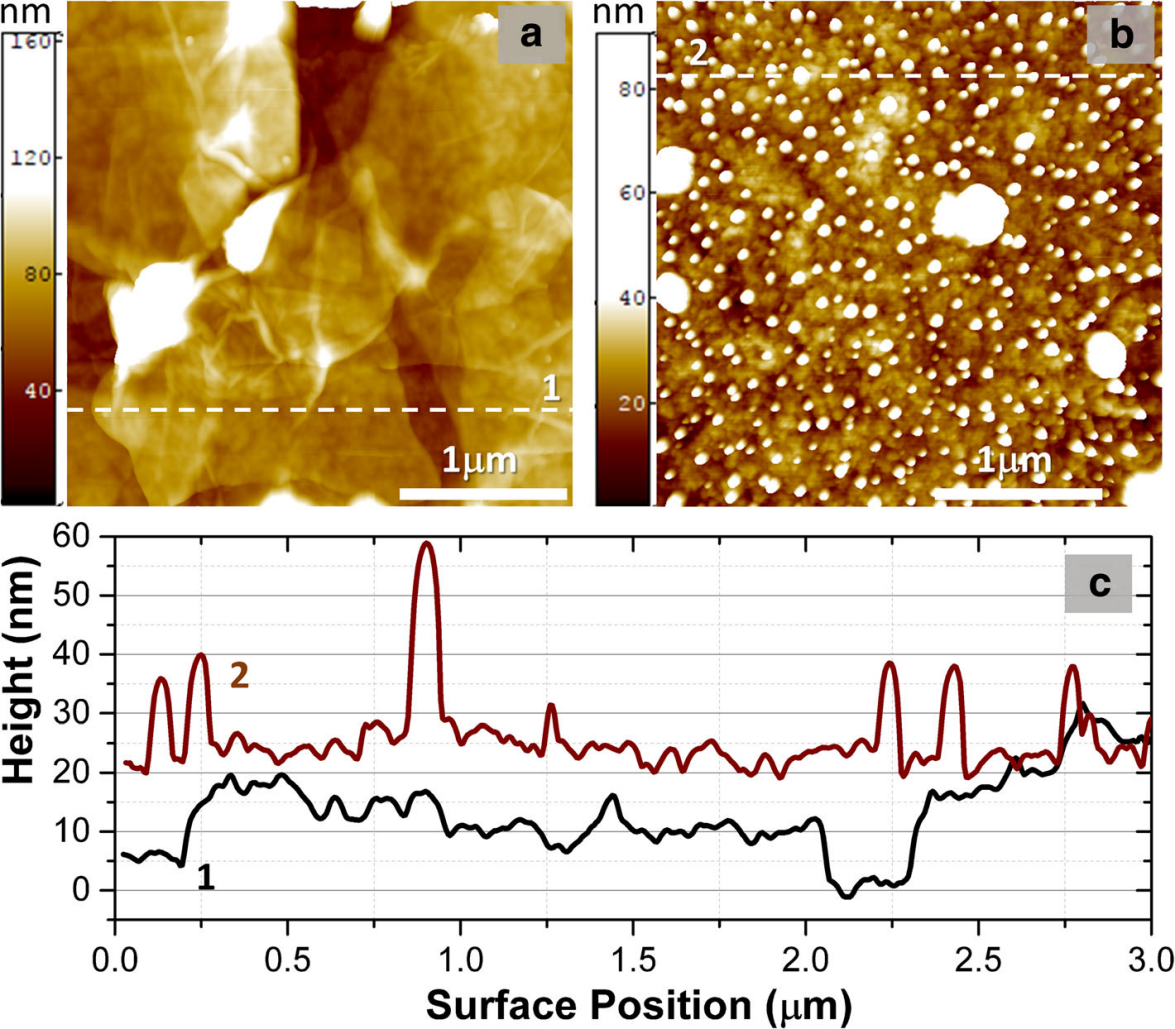
AFM images of drop-casted GO multiflake structures: fragment of clear GO flakes annealed at 110 °C for 15 min (**a**) and fragment of contaminated GO flakes annealed at 180 °C for 15 min (**b**). Corresponding surface height profiles along dashed lines are shown in (**c**)

AFM surface topographic maps of GO and rGO films obtained by drop-casted method are presented in Fig. 6. The films are dense multiflake structures with thickness not less than 30 nm (Fig. 6c). For better estimation of average thickness of our film the thickness was controlled across a drop using the AFM step height profiles of scratches and was equaled to 30–40 nm for ~ 70% of an area of the drop (Additional file 1: Figure S5 (a)). After thermal annealing at 230 °C for 15 min, a thickness of the drop decreases on about 30% (see Additional file 1: Figure S5 (b),(c)). Additionally, in some cases after 180 °C annealing, some nano- and microbubbles are formed on surface of the films (Fig. 6b). Probably, these microbubbles are associated with water molecules desorbed from inner layers of the GO film as well as originated to uncontrolled contaminations in liquid solution of GO.

**Fig. 7:**
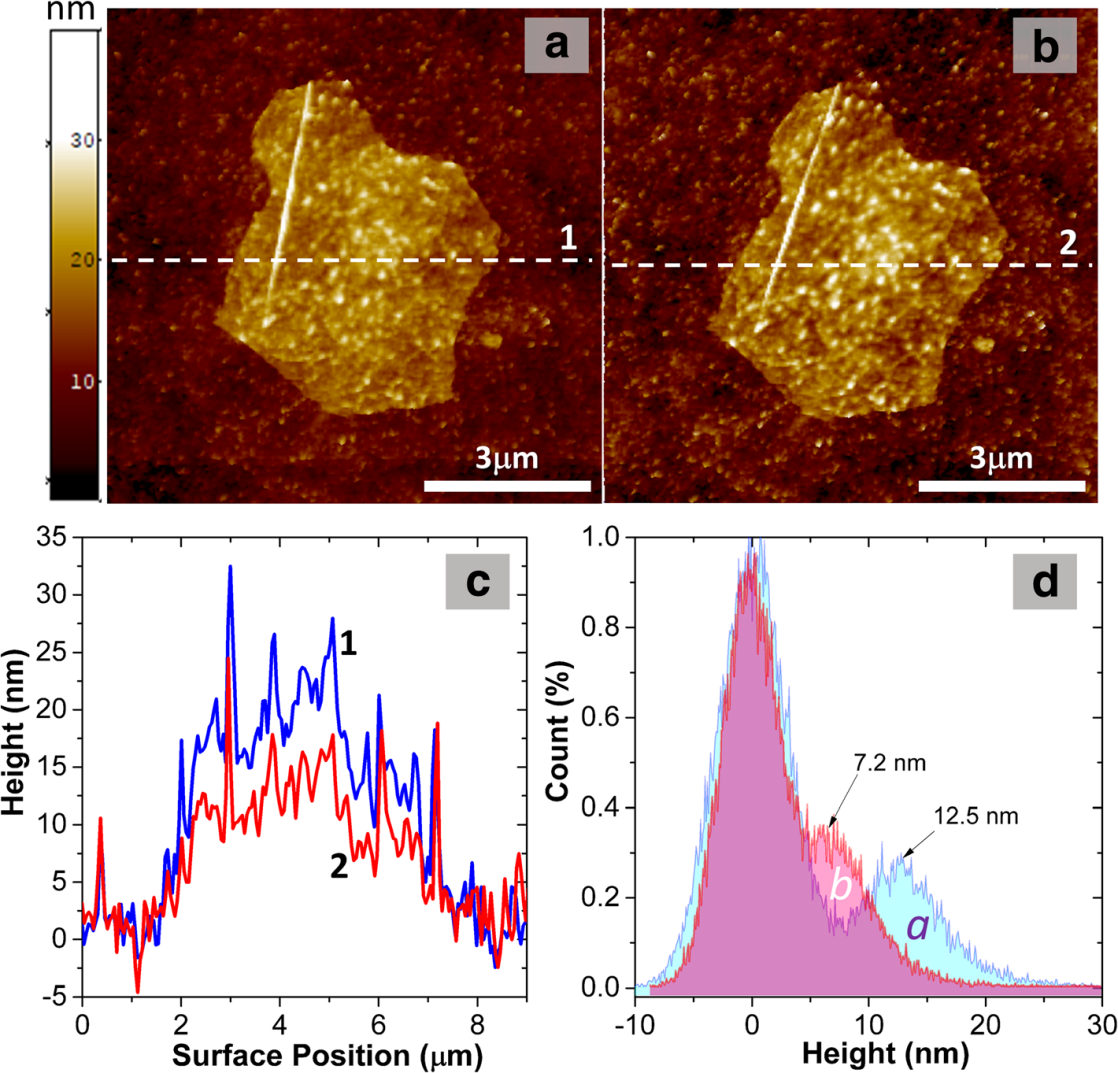
AFM images of single GO flake on the Ni film: initial (**a**) and annealed at 180 °C for 15 min (**b**). Corresponding surface height profiles along dashed lines are shown in (**c**) and height histograms over images are shown in (**d**). Peaks positions according to the peak-fit analysis are marked by arrows

The separate GO flakes deposited on Ni film from the same GO solution were studied by AFM and SKPFM methods to understand better the nature of the GO material transformation during low-temperature annealing. Initial GO flakes have thickness ranging from 8 to 14 nm. The study of the same GO flake after thermal reduction by AFM method allows us to measure a change in thickness and structure topography (Fig. 7). The average thickness of the GO flake is strongly reduced from 12.5 to 7.2 nm for annealing at 180 °C for 15 min that attests desorption of water molecules and oxygen-containing groups from the material. It should be noted that the studied flake consists of several GO layers (about 7–8) that is considerable thinner than the film obtained by drop-cast method.

**Fig. 8:**
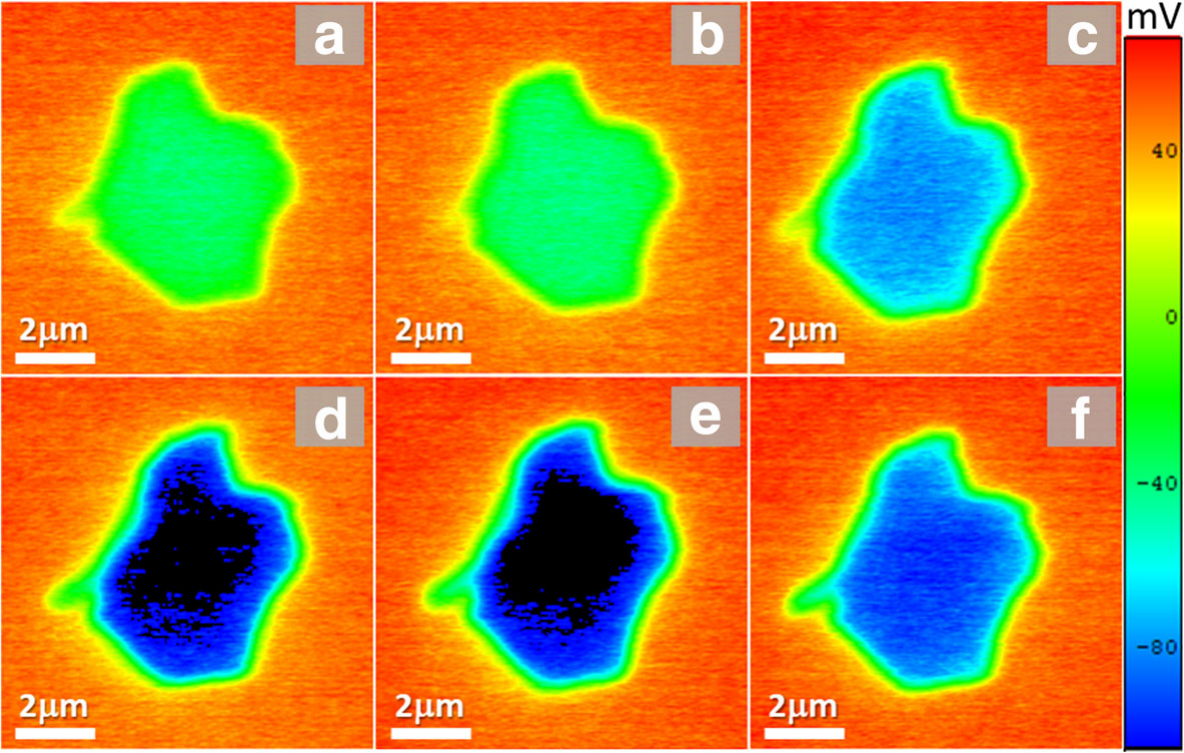
SKPFM maps of the contact potential difference between PtIr tip and GO flake on Ni substrate: initial (**a**) and after annealing for 15 min at 80, 100, 120, 140, and 180 °C (**b**–**f**), correspondingly. Ni substrate used for reference in the SKPFM measurements

**Fig. 9:**
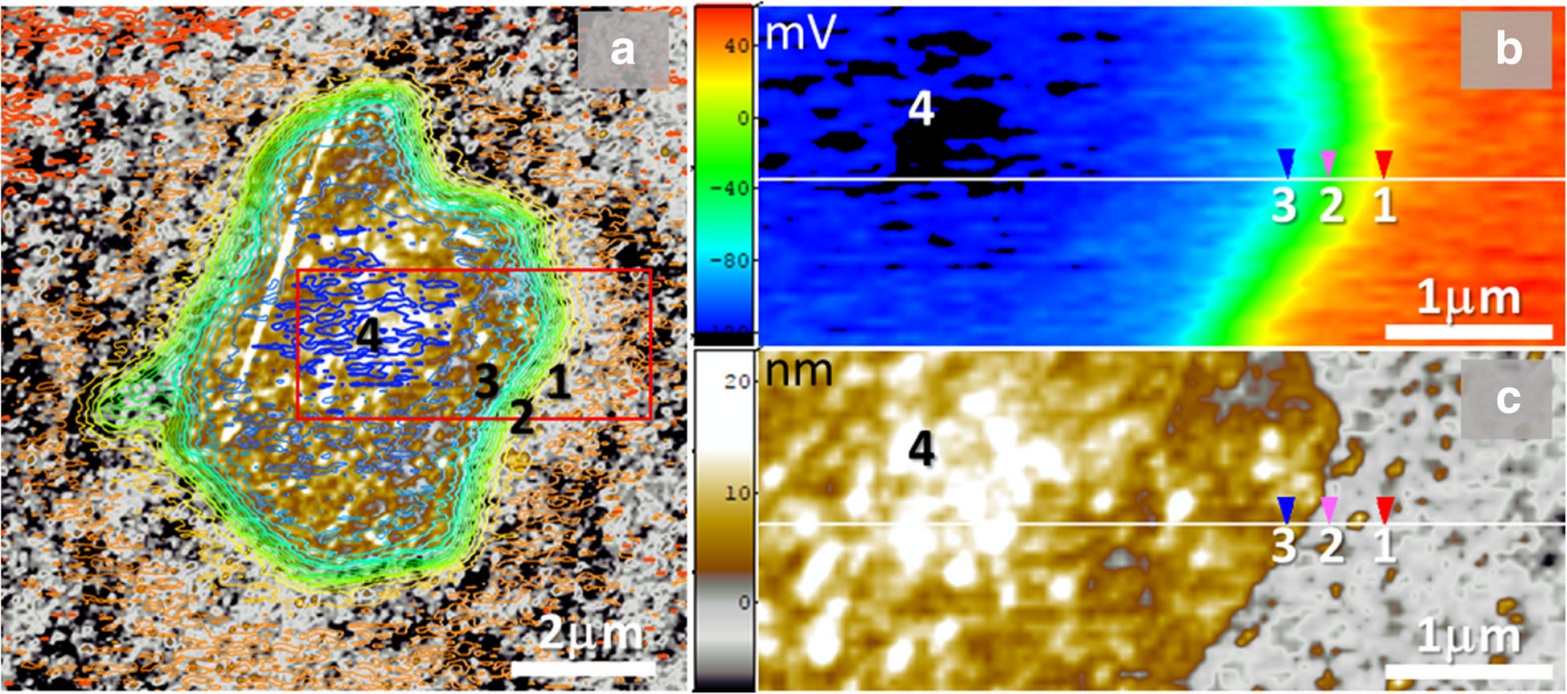
Halo around flake. Topography map overlapped by corresponding SKPFM contour map of the GO flake on Ni substrate annealed at 140 °C for 15 min (**a**). Enlarged maps of surrounding halo are shown in (**b**, **c**). Numbers denote external (#1), external (#2), edge (#3), and intrinsic (#4) zones

Maps of surface contact potential differences (regarding to Ni film) as a function of the annealing temperature are presented in Fig. 8. Several important things should be mentioned. First, the surface contact potential difference (CPD) strongly increases inside the GO flake and reaches the maximum value about 160 mV at 140 °C annealing. Further annealing at higher temperature results in recovery of the CPD. Second, there is a stable halo of CPD around the flake which does not change in its value with increase of the annealing temperature. The halo has three zones—at least two zones (#1 and #2) located out of the flake and one (#3)—at the edge of the flake (see numbers in Fig. 9). It is possible to suggest that external zone #1 is associated with some contaminants accumulated near the edge of flake during the water solvent drying at deposition, zone #2—with electron extraction from Ni into reduced GO flake, whereas the edge zone #3—with stable adsorption of carbonyl groups which desorption requires temperature considerably higher than 220 °C [23] that is totally confirmed by our FTIR and XPS research.

The analysis of reasons which can induce such a strong increase of potential difference inside the flake at temperatures about 120–140 °C leads to conclusion that, probably, this effect is associated with the strong decrease of the work function from nanostructured surface. Such surface is formed during desorption of oxygen epoxy groups together with surface carbon following the chemical reaction [22]:

$$ \mathrm{GO}\to \mathrm{rGO}+\mathrm{CO}\uparrow +{\mathrm{CO}}_2\uparrow +{\mathrm{H}}_2\mathrm{O}\uparrow . $$

Desorption of CO, CO_2_, and H_2_O molecules in the same temperature range was shown by thermally desorption experiments in paper [14]. Generation of large amount of such carbon nanoislands results in a decrease of the average work function of flake area which loses the surface carbon. Subsequent annealing leads to desorption of residual surface carbon decreasing the average thickness of the flake, flattening its surface, and increasing the work function of the surface. The letter factor results in decreasing of average of CPD of the rGO flake surface in respect to Ni potential.

**Fig. 10:**
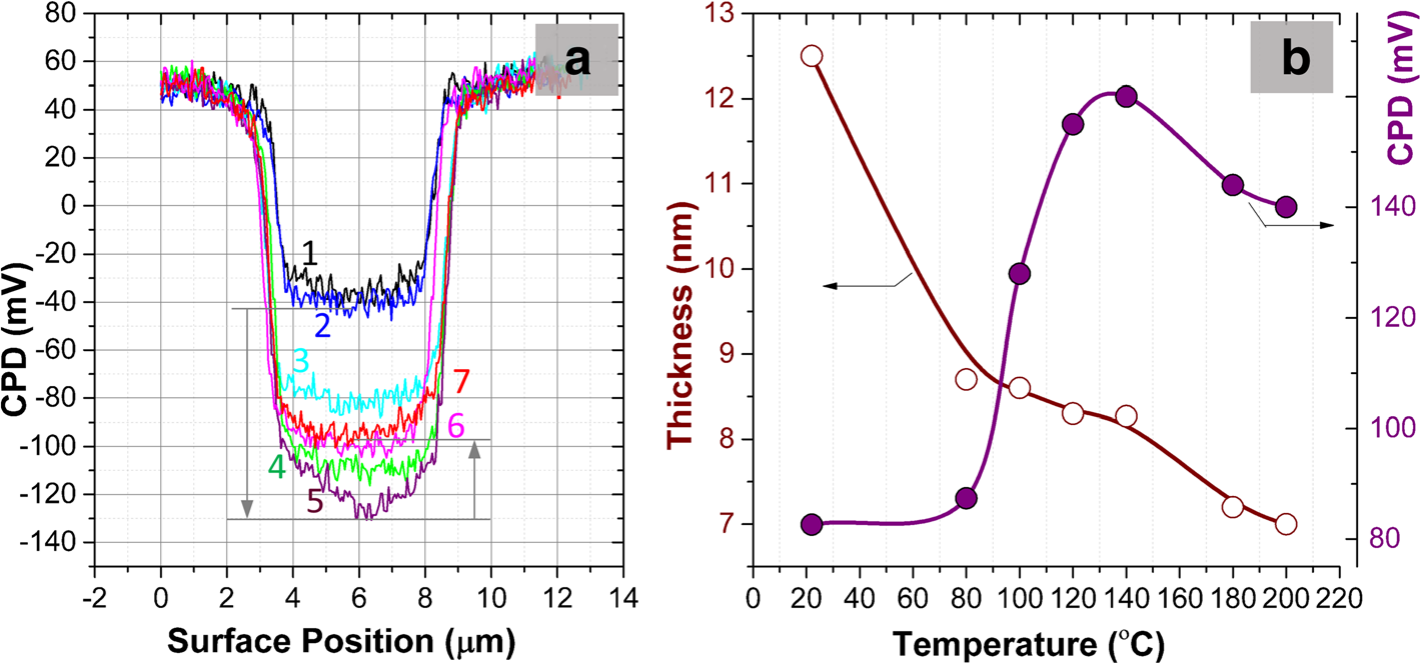
Cross-sections of the SKPFM maps (**a**) shown in Fig. 6. Numbers 1–7 denote cross-sections over initial and annealed samples correspondingly at 80, 100, 120, 140, 180, and 200 °C. Statistically relevant values (from histograms) of flake thickness and contact potential difference between reference Ni film and GO flake are shown in (**b**)

The above described effect can be supported by results presented in Fig. 10. Carbon desorption from the surface flake results in stabilization of the average thickness of the flake and increase of CPD of the rGO in the central area of the flake. Subsequent restoration of the surface decreases the thickness of the flake on thickness of one GO layer (about 1 nm) and decreases CPD. In Fig. 9 in the central area of the flake (zone #4), we can observe protrusions of the material in AFM topological map (Fig. 9c) and the increase of CPD in these places in CPD map (black regions in Fig. 9b).

The comparison of electrical resistivity measurements with AFM and SKPFM ones shows that in both cases, two types of processes are observed: the first one is associated mainly with molecular interlayer water desorption, and the second process—with desorption of oxygen epoxy groups together with carbon atoms. These processes in resistivity and AFM and SKPFM measurements are manifested at different annealing temperature. The first process appears in resistivity measurement up to 150 °C, whereas for AFM and SKPFM measurements the process takes place up to 100–110 °C. First of all, such temperature difference is related to different locations of the studied process. The electrical measurements are integral and determined by total thickness of thick (about 40 nm) GO film, whereas the AFM and SKPFM measurements are the surface ones. Additionally, in paper [42], it was shown that the process of water desorption from inner GO layers is noticeably difficult and will appear at higher temperature in thicker film.

## Conclusions

Performed research of thermal reduction of GO in air ambient has shown that the low-temperature annealing up to 250 °C allows to strongly decrease the GO film’s resistivity (about seven orders). In the studied range of the annealing temperatures, two main processes take place. The first process is molecular water and bonded OH groups desorption with strong reduction of the film thickness, the second process is controlled by epoxy and alkoxy oxygen desorption with destruction of carbon basal plane that considerably reduces the GO work function. Enhanced temperature (180–200 °C) cleans the surface from carbon nanoinclusions recovering the rGO work function and thinning the film. Resistivity of the reduced GO film is stable, unchanging strongly during 6 months.

## Additional file

Additional file 1: **Figure S1.** SKPFM maps (a–d) of single GO flake on the Ni film: initial (a, c) and annealed at 120 °C for 15 min (b, d) for lift height of 20 nm (a, b) and 40 nm (c, d). Figure S2. CPD maps of dried (120 °C, 15 min) GO flakes on Ni substrate: sample grounded (a) and tip grounded cases (b). Topography image is also shown (c). Figure S3. Areas for C1s and O1s peaks calculated for samples annealed at 50 °C, 120 °C, 180 °C, and 250 °C (from top to bottom correspondingly). Figure S4. Fitting of Raman spectrum for GO annealed in air at 140 °C for 15 min by five bands as proposed in paper [1 s]. Figure S5. Typical thickness of rGO film (a), AFM integral result. The decreasing of film effective thickness under annealing was estimated using a clear rapper on surface. The thickness decreased, in the measured point, from 93 nm (b) to 62 nm after annealing at 230 °C for 15 min (c). (DOC 3483 kb)
