# Supplementary material for: Low-Temperature Reduction of Graphene Oxide: Electrical Conductance and Scanning Kelvin Probe Force Microscopy
Source: Nanoscale Res Lett. 2018 May 8;13:139. doi: 10.1186/s11671-018-2536-z (PMC5940978; doi:10.1186/s11671-018-2536-z)
Supplement: Supplementary file 1 — Figure S1. SKPFM maps (a–d) of single GO flake on the Ni film: initial (a, c) and annealed at 120 °C for 15 min (b, d) for lift height of 20 nm (a, b) and 40 nm (c, d). Figure S2. CPD maps of dried (120 °C, 15 min) GO flakes on Ni substrate: sample grounded (a) and tip grounded cases (b). Topography image is also shown (c). Figure S3. Areas for C1s and O1s peaks calculated for samples annealed at 50 °C, 120 °C, 180 °C, and 250 °C (from top to bottom correspondingly). Figure S4. Fitting of Raman spectrum for GO annealed in air at 140 °C for 15 min by five bands as proposed in paper [1 s]. Figure S5. Typical thickness of rGO film (a), AFM integral result. The decreasing of film effective thickness under annealing was estimated using a clear rapper on surface. The thickness decreased, in the measured point, from 93 nm (b) to 62 nm after annealing at 230 °C for 15 min (c). (DOC 3483 kb) [file 11671_2018_2536_MOESM1_ESM.doc]

**Additional file 1**

Low-Temperature Reduction of Graphene Oxide: Electrical Conductance and Scanning Kelvin Probe Force Microscopy

^1)^ O.M. Slobodian, ^2)^P.M. Lytvyn, ^2)^A.S. Nikolenko, ^2)^V. M. Naseka, ^3)^O.Yu.Khyzhun,

^1,2)^A.V. Vasin, ^4)^S.V. Sevostianov, ^1,2)^A.N. Nazarov

^1)^National Technical University of Ukraine "Igor Sikorsky KPI", Kyiv, Ukraine

^2)^V. Lashkaryov Institute of Semiconductor Physics NAS of Ukraine, Kyiv, Ukraine

^3)^ Frantsevych Institute for Problems of Materials Science NAS of Ukraine, Kyiv, Ukraine

^4)^Chuiko Institute of Surface Chemistry NAS of Ukraine, Kyiv, Ukraine

* Corresponding authors: O.M. Slobodian, [a.slobodyan-2015@kpi.ua](mailto:a.slobodyan-2015@kpi.ua)

**(a)**
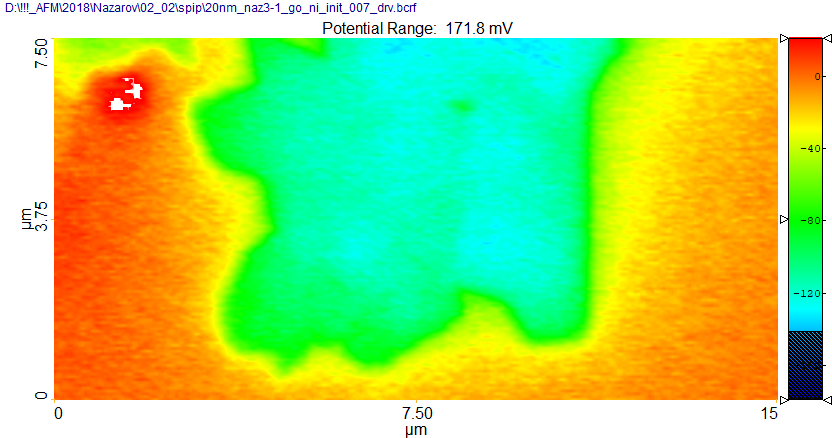
  **(b)
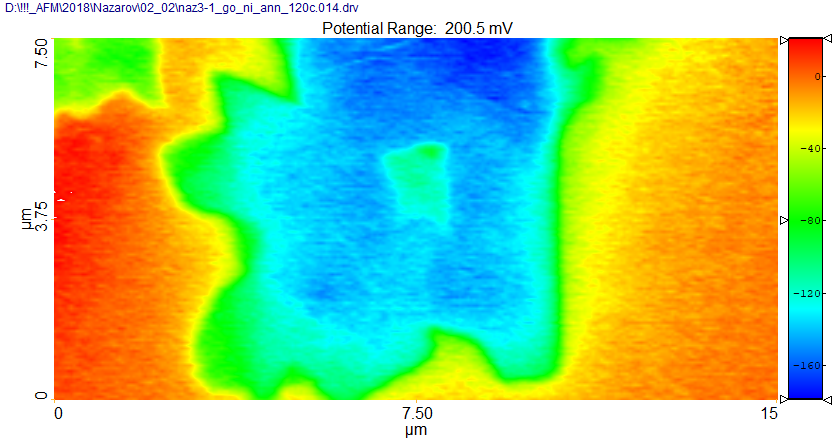
**

**(c)**
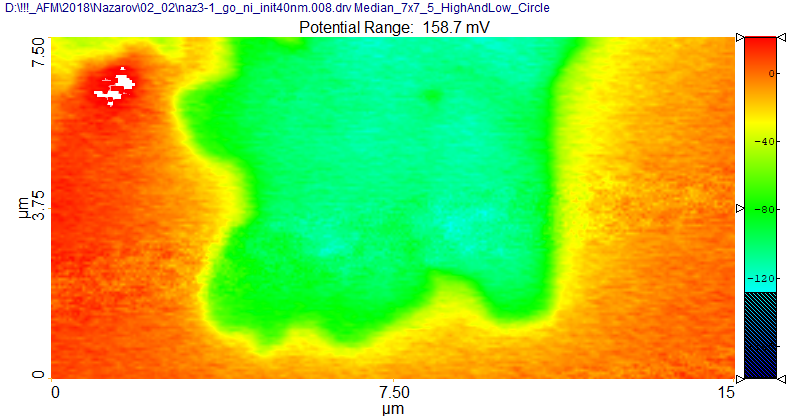
 **(d)**
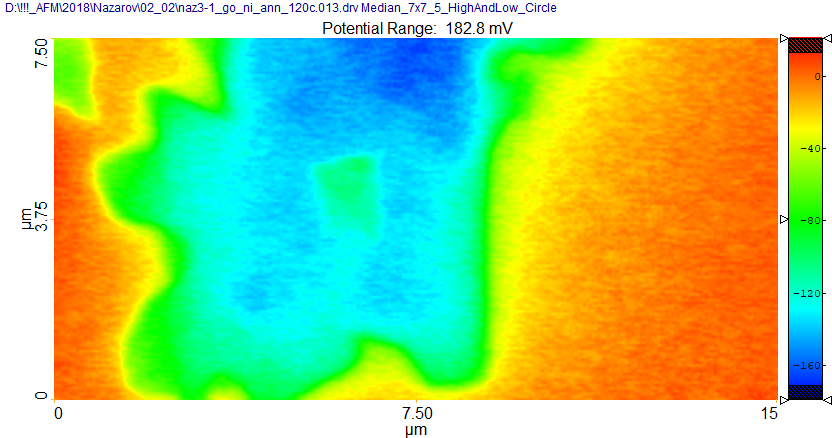


Figure S1. SKPFM maps (a-d) of single GO flake on the Ni film: initial (a, c) and annealed at 120°C for 15 minutes (b, d) for lift height of 20 nm (a, b) and 40 nm (c, d).


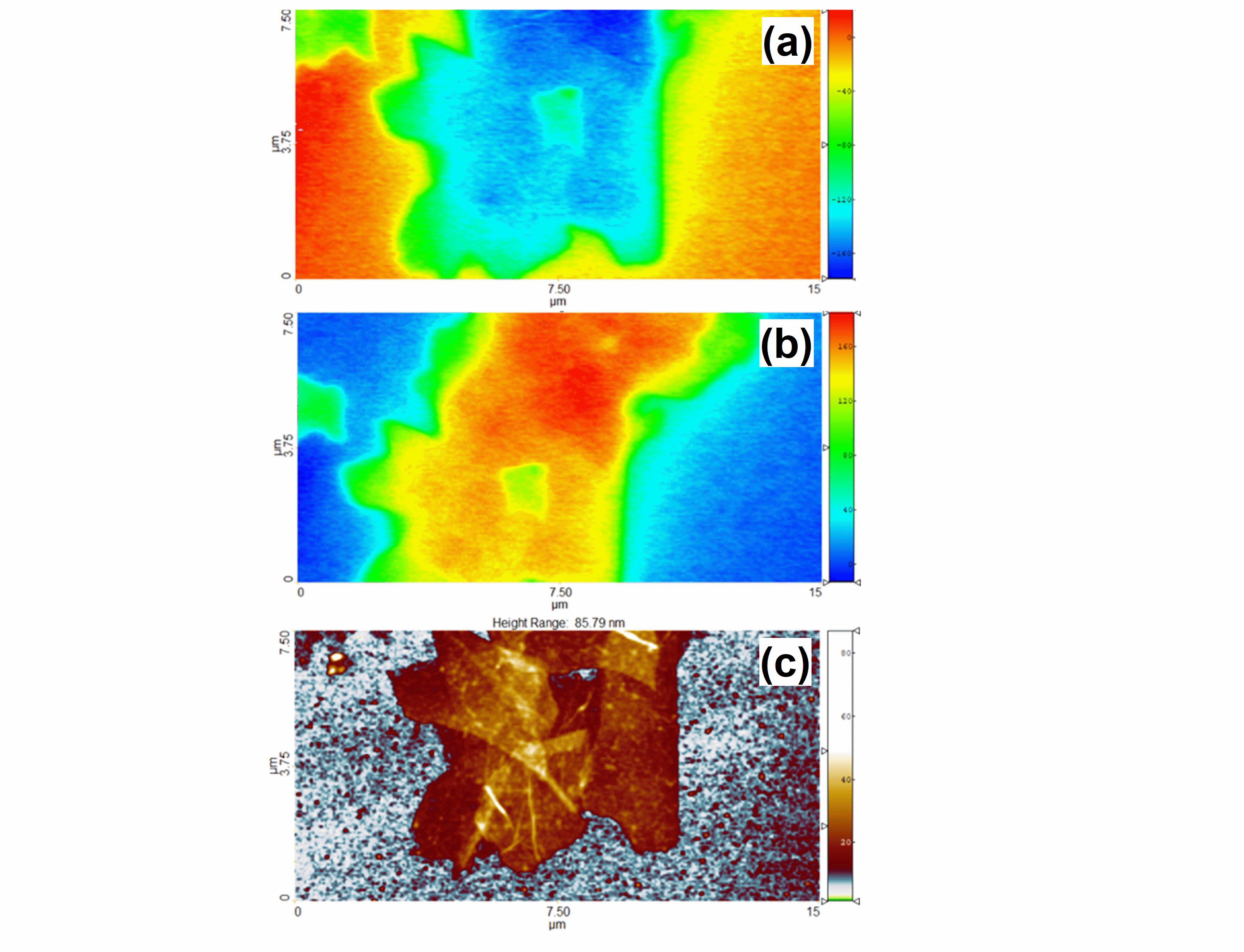


Figure S2. CPD maps of dried (120°C, 15min) GO flakes on Ni substrate: sample grounded (a) and tip grounded cases (b). Topography image is also shown (c)


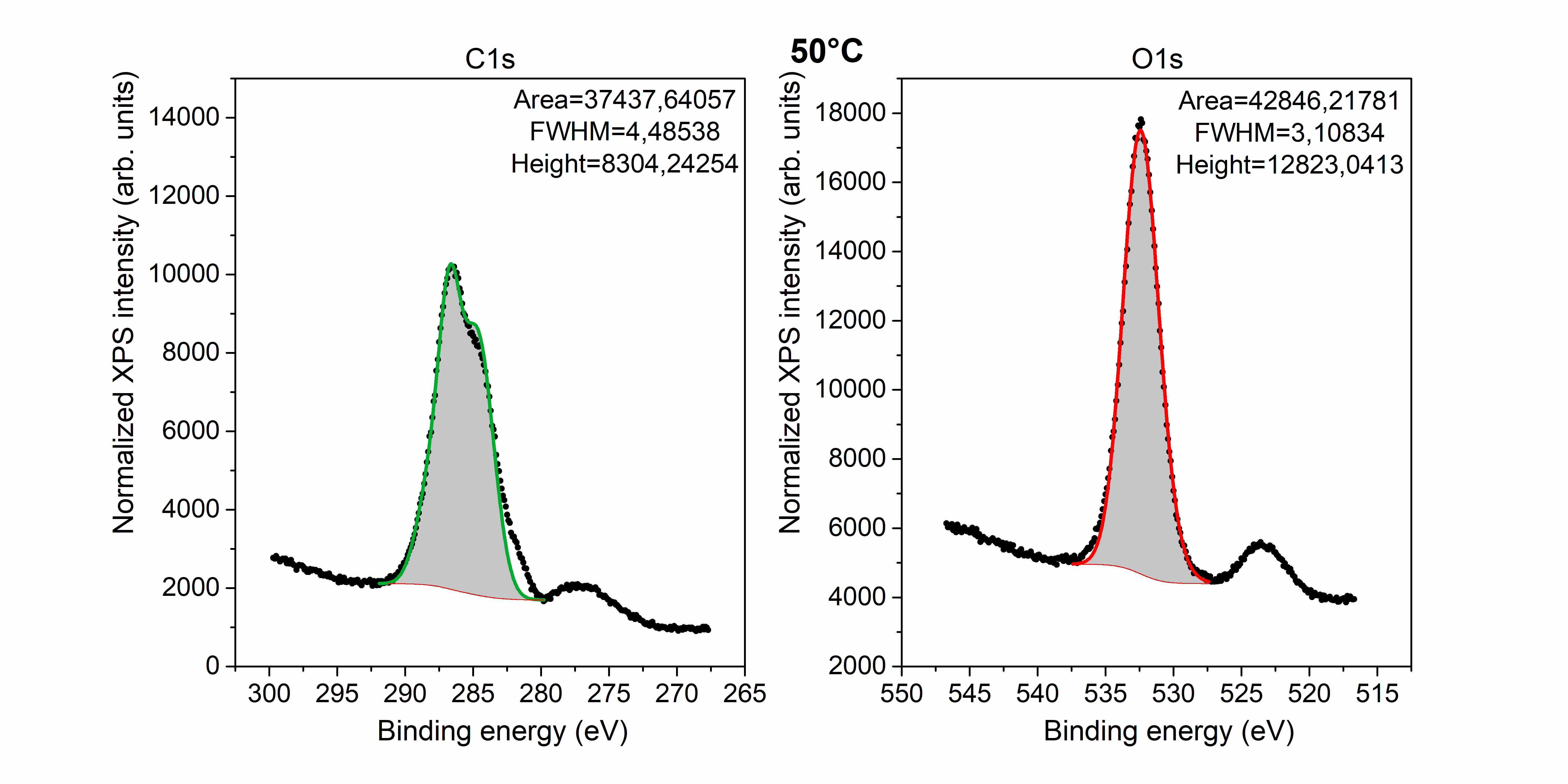


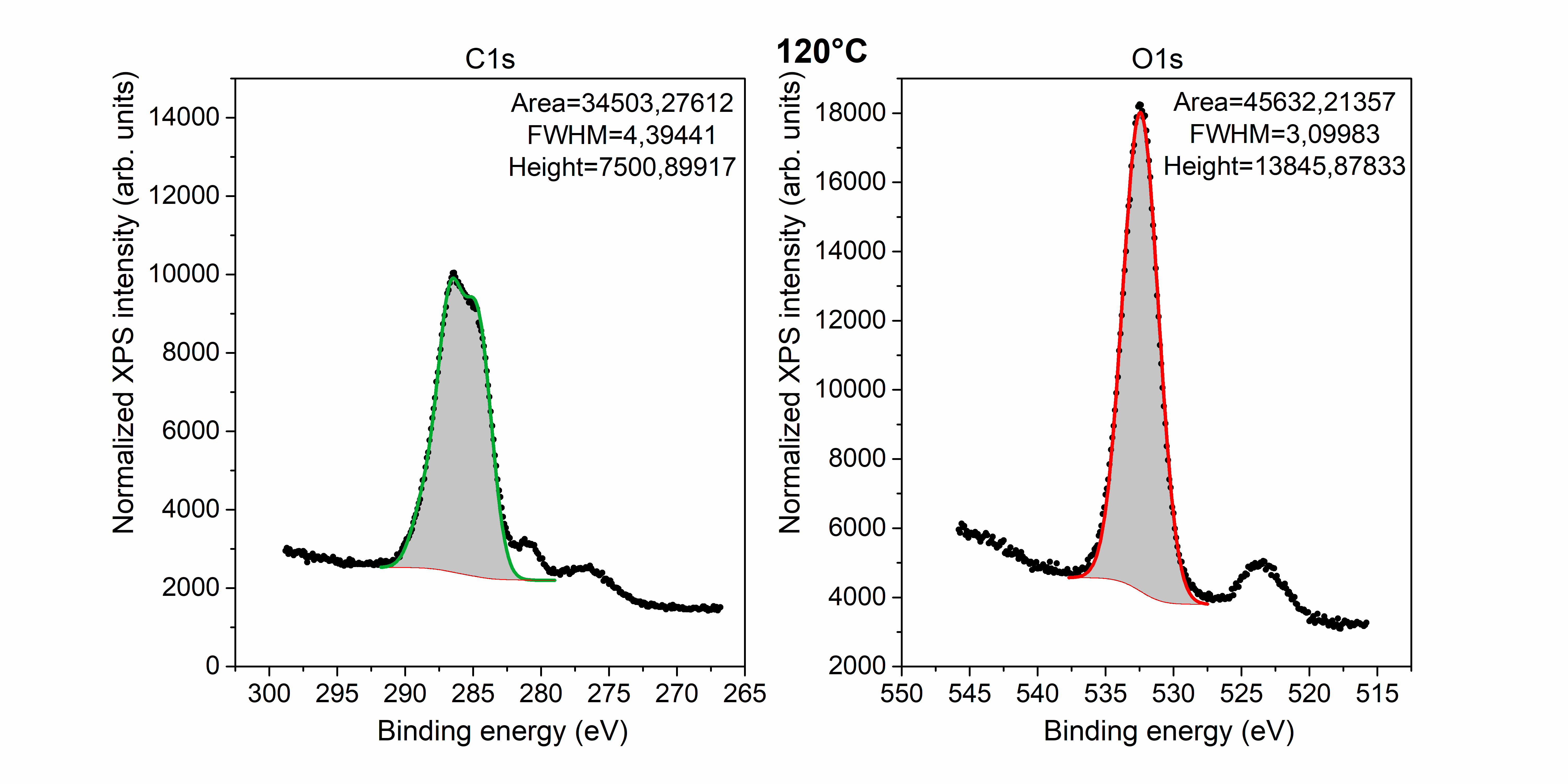


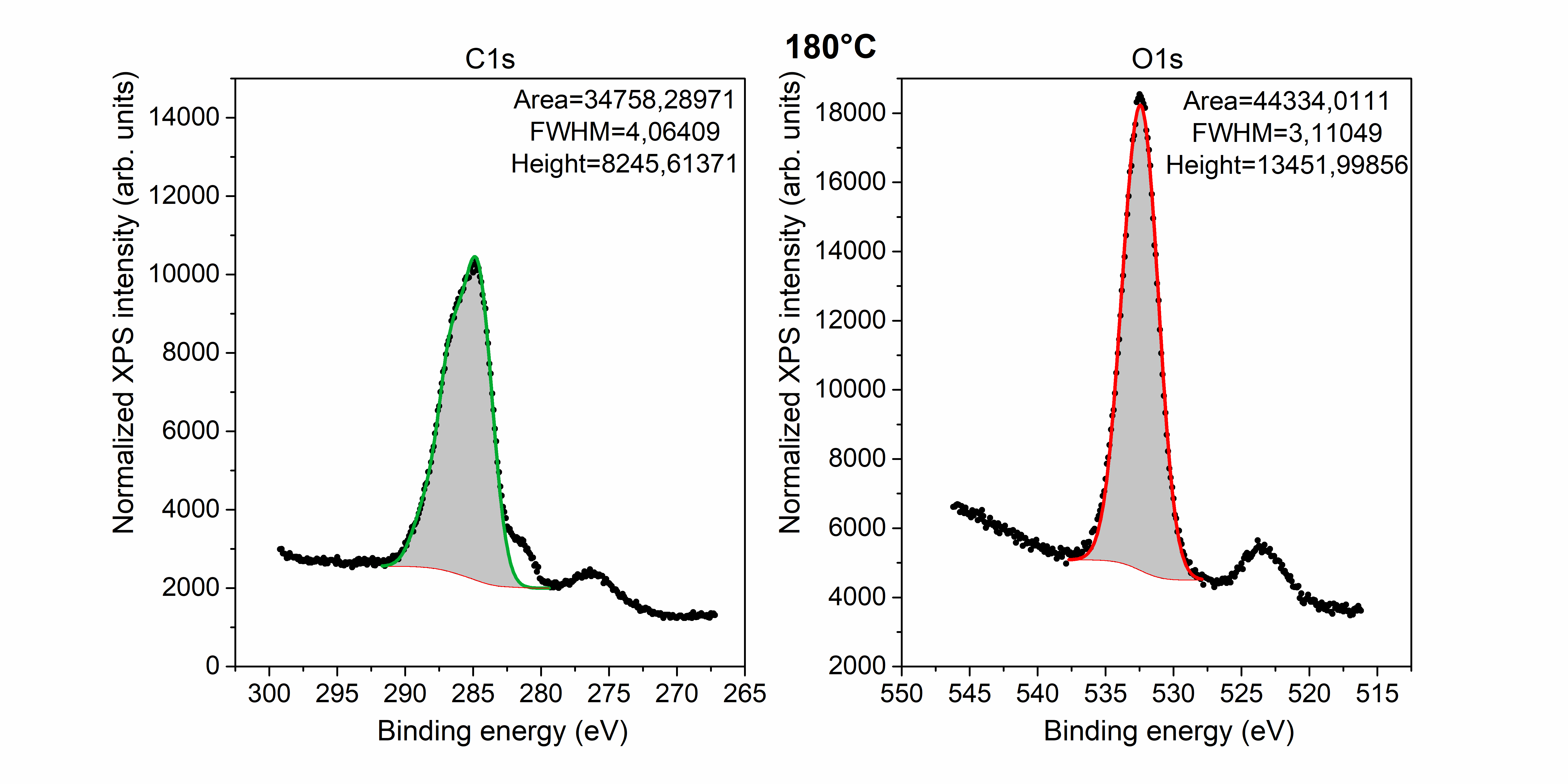


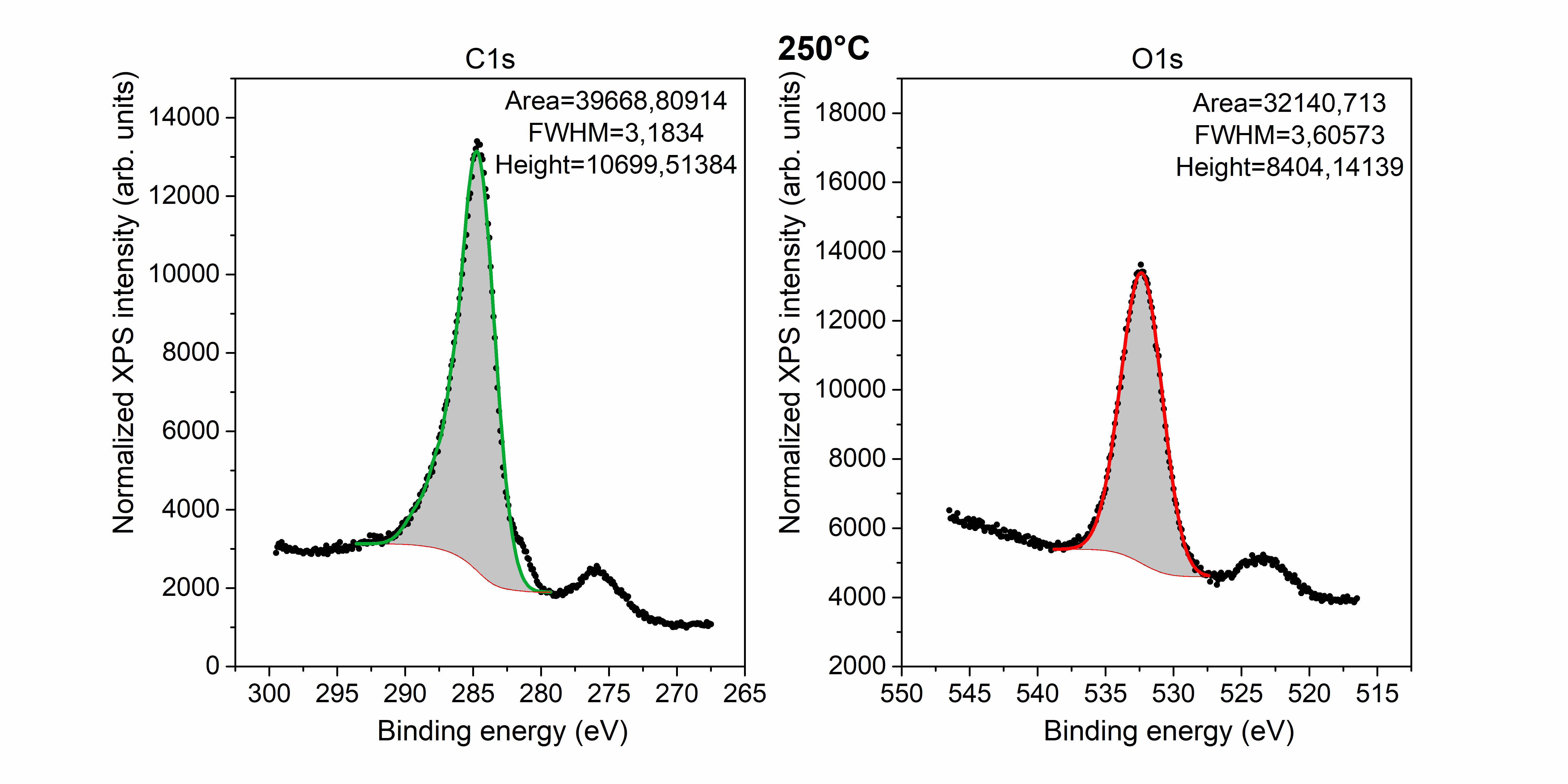


Figure S3. Areas for C1s and O1s peaks calculated for samples annealed at 50°C, 120°C, 180°C and 250°C (from top to bottom correspondingly)


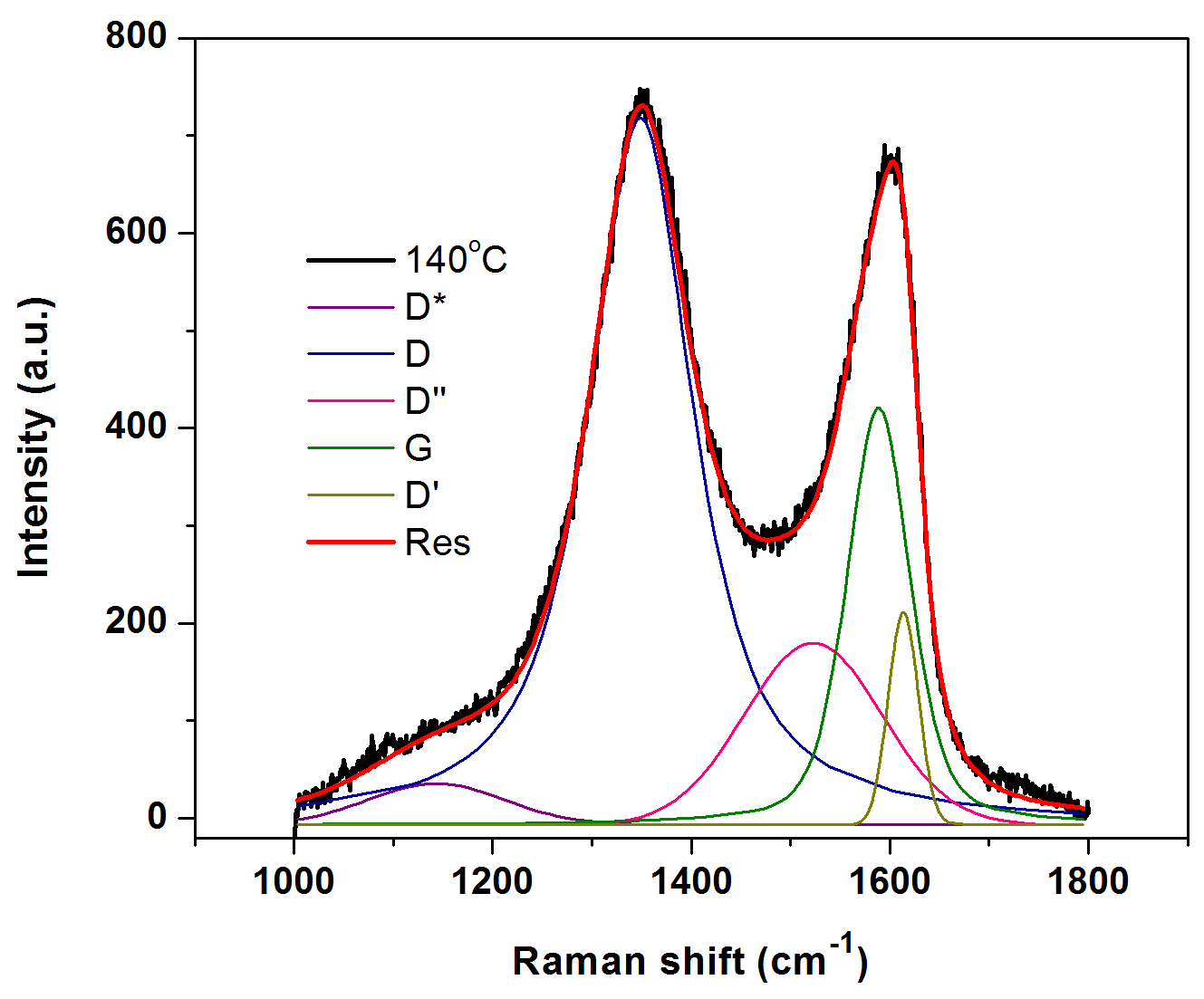


Figure S4. Fitting of Raman spectrum for GO annealed in air at 140°C for 15 min by five bands as proposed in paper [1s].


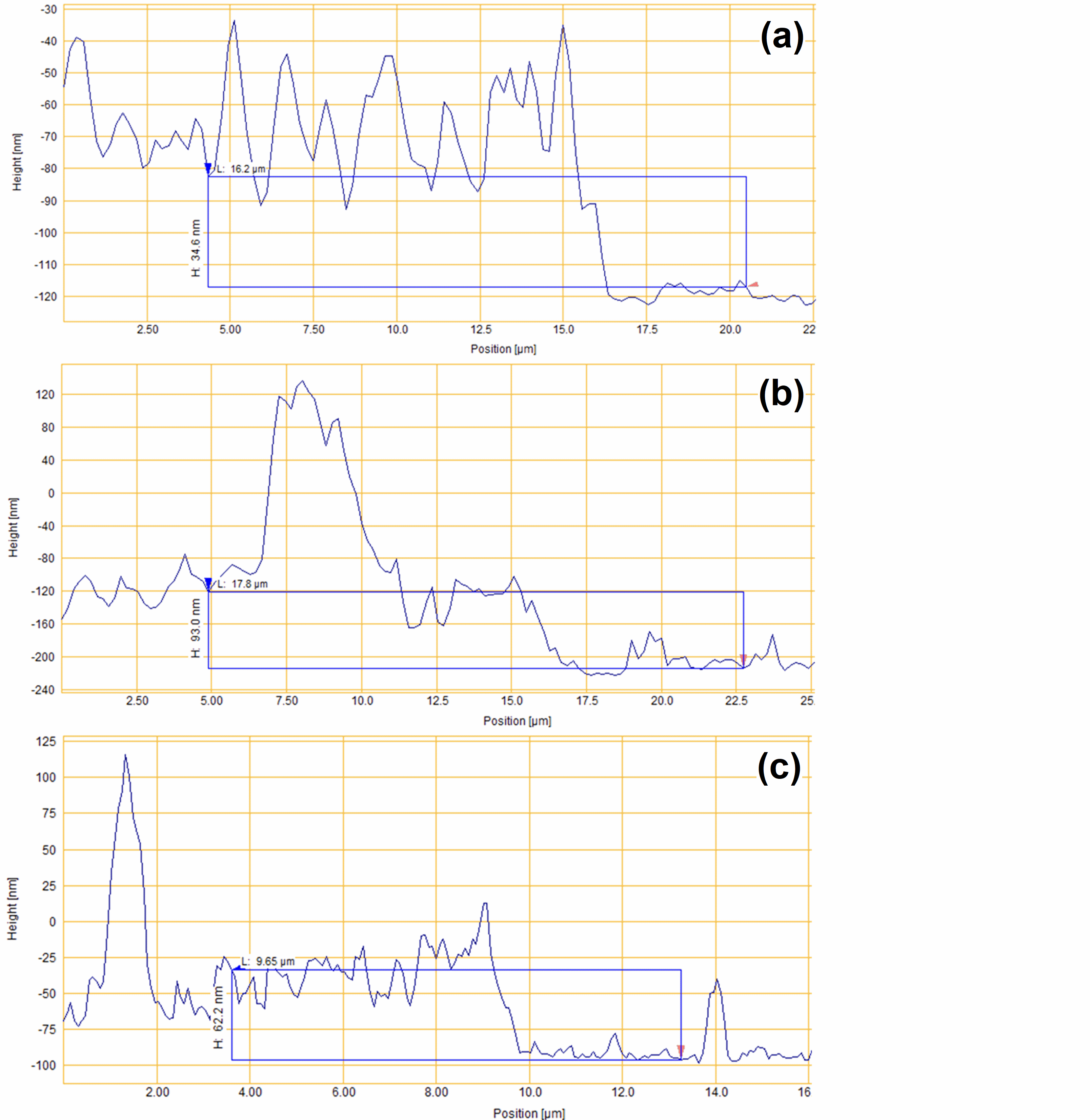


Figure S5. Typical thickness of rGO film (a), AFM integral result. The decreasing of film effective thickness under annealing was estimated using a clear rapper on surface. The thickness decreased, in the measured point, from 93nm (b) to 62nm after annealing at 230°C for 15min (c)

References

[1s] Claramunt S, Varea A, López-Díaz D, et al. (2015) The importance of interbands on the interpretation of the Raman spectrum of graphene oxide. J. Phys. Chem. C 119: 10123-10129.
